# Impact of musculoskeletal symptoms on physical functioning and quality of life among treated people with HIV in high and low resource settings: A case study of the UK and Zambia

**DOI:** 10.1371/journal.pone.0216787

**Published:** 2019-05-13

**Authors:** Nikolien S. Van de Ven, Owen Ngalamika, Kevin Martin, Kevin A. Davies, Jaime H. Vera

**Affiliations:** 1 Brighton and Sussex University Hospitals NHS Trust, Brighton, United Kingdom; 2 University Teaching Hospital, Lusaka, Zambia; 3 Department of Medicine, Brighton and Sussex Medical School, Brighton, United Kingdom; 4 Department of Global Health and Infection, Brighton and Sussex Medical School, Brighton, United Kingdom; Imperial College London, UNITED KINGDOM

## Abstract

**Background:**

Musculoskeletal symptoms in people living with HIV (PLWH) such as pain, joint stiffness, and fatigue are commonly reported. Prevalence rates of up to 45%, 79% and 88% respectively have been reported. However, very little is known about differences in prevalence and impact of musculoskeletal symptoms on physical functioning and quality of life of PLWH on effective combined antiretroviral treatment (cART) in high and low-resource settings.

**Methods:**

A cross-sectional study of PLWH on effective cART enrolled from two large urban clinics in the UK and Zambia was conducted in 2016. Eligible participants had no history of trauma to the joints within 4 weeks of recruitment, or documented evidence of previous rheumatic disease. Current musculoskeletal symptoms, functional ability, and health-related quality of life were evaluated using the health assessment (HAQ) and quality-of-life short form (SF-36) self-reported questionnaires.

**Results:**

214 patients were enrolled (108:UK and 106:Zambia). Participants from Zambia were younger (47 vs 44 years) and had significantly lower CD4 counts (640 vs 439 cells/mL p = 0.018) compared to those from the UK, while the UK group had lived with HIV longer (11 vs 6 years; p<0.001) and reported more comorbidities than the Zambian group (66% vs 26%; p<0.001). Musculoskeletal pain was common in both groups (UK:69% vs Zambia:61% p = 0.263) but no significant differences in physical functional capacity between the groups were observed. However, the UK group had significantly worse quality of life measurements (general health, vitality, mental health, emotional, and social functioning) associated with musculoskeletal symptoms compared to the Zambian group (p<0.001).

**Conclusions:**

Musculoskeletal symptoms in PLWH from both the UK and Zambia were common. PLWH in the UK reported worse quality of life measures associated with musculoskeletal symptoms compared to those in Zambia, suggesting that factors such as mental health, patient expectations and multimorbidity might play a role in determining well-being and quality of life of PLWH with musculoskeletal symptoms.

## Introduction

The introduction of combination antiretroviral treatment (cART) has meant that people living with HIV (PLWH) in many parts of the world can now expect to experience a near-normal life expectancy, providing a good CD4 cell count response and undetectable viral load are achieved [1, 2]. However, due to the availability of cART the cohort of PLWH globally is ageing and therefore the prevalence of age-associated co-morbidities such as cardiovascular disease, chronic kidney disease, osteoporosis and non-AIDS malignancies are expected to increase [3].

Before cART musculoskeletal symptoms such as pain were a common symptom of end-stage HIV, but there is growing evidence that musculoskeletal symptoms including arthralgia, myalgia, and soft tissue rheumatism are common in PLWH on effective cART in the absence of established rheumatological disease [4, 5]. Prevalence of musculoskeletal symptoms among HIV-infected adults has been reported to be between 10% to 45% in both low and high resource settings [4, 6–8]. Pain has been reported to be one of the most common physical symptoms among PLWH, with reported prevalence ranging from 30% to 83% [6, 9]. Musculoskeletal symptoms among PLWH have both clinical and public health importance. Not only are symptoms such as musculoskeletal pain associated with psychological distress, emotional problems, poor quality of life and suicidal ideation [10, 11], but they are also known to impact on activities of daily living [12] and are associated with higher rates of sick leave, loss of employment and lower satisfaction with health care [13]. Among PLWH, pain is also associated with poorer adherence to cART [14] and missed clinic visits [15].

Whilst pain has been identified as a prevalent problem in PLWH, other musculoskeletal symptoms have been largely neglected in clinical practice, and little research has been conducted on this area either in low and high-income settings in the modern ART era among individuals in whom HIV infection is largely controlled. With the aim to determine independently the prevalence and impact of musculoskeletal symptoms in two countries which are culturally and demographically different in terms of the HIV epidemic, we report the prevalence and impact of these symptoms on quality of life and physical activity among a group of PLWH in the United Kingdom and Zambia. We further compare both groups in an exploratory way to determine first if there are differences and second the type of variables where those differences are found to inform any future studies and interventions.

## Materials and methods

### Study design and setting

A cross-sectional study of PLWH on effective cART enrolled from two large urban clinics in the UK and Zambia was conducted. Participants from Zambia were recruited from a large clinic at the University Teaching Hospital (UTH) in Lusaka where HIV care is provided according to the national guidelines [16]. Participants from the UK were recruited from a clinic in Brighton. Several factors make Lusaka and Brighton ideal for conducting this research. The UTH is the largest hospital in Zambia with a dedicated HIV department treating many PLWH daily. Brighton has the largest HIV population outside of London, with a prevalence of 7.96/1000 of the general population aged 15–59 [17] and a single clinic where 90% of the local diagnosed population attend for HIV care.

Assuming a 45% prevalence estimate of musculoskeletal symptoms, based on an average prevalence calculated from previous research papers [4, 6–8], the total sample size of 105 participants from each location is required with a desired precision of 8% and 90% confidence level. This level of confidence and sample size was deemed sufficient for the scope of this small-scale cross-sectional survey. Our study was not powered to determine a clinically important difference in the prevalence of symptoms in the two countries.

Ethics approval was obtained from the Biomedical Research Ethics Committee of the University of Zambia, the Research and Ethics and Governance Committee in Wales and the UK Health Research Authority (HRA); Reference: 16/WA/0285. Written informed consent was obtained from all patients.

### Participants and inclusion and exclusion criteria

Eligible participants were >18 years of age, with no documented prior history of rheumatological disease. Participants with a known rheumatic comorbidity were thus excluded as the aim off the survey is to assess the prevalence of rheumatic-type symptoms in PLWH not known to have a rheumatic disease. Participants with a history of trauma to the joints within 4 weeks of recruitment were excluded. ‘Four weeks’ no history of trauma to the joints was deemed sufficient time for a possible trauma to the joints to have resolved and therefore would not attribute to the reasoning behind a participant experiencing musculoskeletal symptoms.

Participants who consented to participate completed a series of paper-based self-reporting questionnaires evaluating musculoskeletal symptoms, functional ability, and health-related quality of life. A symptom was defined as a subjective loss of normal function or feeling which was recognized by the person experiencing them. All questionnaires were translated into Bemba and Nyanja (Zambia’s most common languages) and facilitated by a research assistant fluent in both languages who was able to explain in detail how to complete the questionnaires if needed (see S2, S3, S4, S6, S7, S8 and S9 Files for questionnaires used in Bemba, Nyanja and English).

### Outcome measures

#### Assessment of musculoskeletal symptoms

A questionnaire, previously validated amongst PLWH for its usefulness in identifying the presence, type and frequency of musculoskeletal symptoms [18] was used to collect the data, containing questions on symptoms such as muscle and joint pain, joint swelling, joint movement, and fatigue. Other symptoms involving the skin, eyes, and mouth were also documented. Pain and fatigue were evaluated using a 10-point numeric visual analogue scale from 0 to 10 (0 = no symptoms, 10 = severe symptoms) of how much pain or fatigue participants experienced S9 File [19, 20].

#### Assessment of physical functioning

Physical functioning was evaluated using the Stanford Health Assessment Questionnaire (HAQ), an instrument originally developed for rheumatology to assess patient’s capacity to perform activities of daily living [21, 22] but has since been validated and applied to PLWH across diverse disciplines and various cultures [23, 24].

The HAQ queries the ability to perform 20 activities of daily living divided into 8 categories. The ability to perform each activity has four possible responses (0 = no difficulty, 1 = some difficulty, 2 = much difficulty, to 3 = unable to do). The total HAQ score is calculated from the mean of the scores for each category as previously reported [25].

To adapt the HAQ culturally, modifications of individual items are sometimes necessary. These have proved equally as reliable and valid as their parent [26]. If there were any types of items that needed adaptation an appropriate substitution in keeping with the original intent of the item was made by the research assistant.

#### Assessment of quality of life

Health-related quality of life (QoL) was measured using the quality of life short form (SF-36). The SF-36 is a generic measure of QoL that has been widely validated and noted to be reliable for use across a range of healthcare professions, settings, and patients including PLWH [27–31]. It measures QoL across eight emotional and physical domains: physical functioning; role limitations due to physical health; role limitations due to emotional problems; energy/fatigue; emotional well-being; social functioning; pain; general health. In the SF-36 a score of 0 represents the worst possible QoL, and 100 indicates full QoL.

#### Variables

All information regarding the patient‘s sociodemographic characteristics such as age and years since diagnosis (years), gender (female or male), education (primary, secondary, college, university) smoking habits (smoker or non-smoker), work status (employed or unemployed), number of comorbidities (>2 or <2), on cART (yes or no) and type of cART (protease inhibitor-based therapy, integrase-inhibitor based therapy, NNRTI-based therapy, complex regimen >1 of the aforementioned) was collected using a specific form designed for this research (see S1, S5 and S10 Files). Clinical test results including current CD4 count (cells/mm^3^) and viral load (>40 copies/ml or <40 copies/ml defined as an undetectable viral load) were recovered for each patient.

Any additional clinical and sociodemographic data was obtained from the medical notes.

### Statistical analysis

Differences in baseline demographics, clinical and HIV parameters between the UK and the Zambia cohorts were determined using Chi-square, Fisher’s exact test, *t*-test or Mann-Whitney test depending on data–set size and type and normality of the data. Multiple linear regression models were used to predict three SF-36 domains (general health, vitality and mental health). The other domains were not predicted due to heavily skewed data. The models used demographic factors (age, gender, smoking status, presence of co-morbidities, work status and CD4 count) and musculoskeletal symptoms (presence of reduced joint movement, presence of morning joint or muscle stiffness, muscle pain severity and fatigue severity). Of note, the P-P plots for the Zambia general health and mental health models suggest that the assumption of normality for the residuals may have been violated. However, these are mild deviations and so the results are likely still valid. Participants not on ART were excluded from the regression analyses. All statistical analyses were performed using SPSS (IBM Version 24).

## Results

### Demographics and clinical characteristics

108 participants in the UK and 106 in Zambia were enrolled. Table 1 describes the demographics and clinical characteristics for both study groups. Participants from the UK were mainly male, men that have sex with men, slightly older (mean age 47 vs 44 years old), had been living with HIV for longer (11 vs 6 years), and were more likely to have more than 2 comorbidities (66% vs 26%) compared to the Zambian participants. Zambian participants were mainly female, heterosexual, less likely to smoke (6% vs 32%) and had lower median CD4 T cell counts than the UK participants (439 vs 640). Only 3 participants from Zambia were not on cART, with 100% of those in the UK group having had a viral load of <40 copies/mL. There was a significant difference between the cART regimes used, with 85% of patients in the Zambian cohort and 47% of the UK group taking NNRTI-based cART including efavirenz and nevirapine (mainly in Zambia) and rilpivirine in the UK. All participants completed the study questionnaires.

**Table 1:** Baseline characteristics.

Characteristics	Brighton (*n* = 108)	Zambia (*n* = 106)	p-value
Age, mean (SD), years	47.6 (11.5)	44.1 (12.3)	0.034
Years since diagnosis, median (range), years	11.5 (0–35)	6 (0–40)	0.001
Gender, *n* (%)			
Female	11 (10.2)	58 (54.7)	0.001
Male	97 (89.8)	48 (45.3)	
Education, university-level education *n* (%)	41 (38.0)	12 (11.4)	0.001
Work status, unemployed *n* (%)	41 (38.3)	60 (56.4)	0.017
Smoker, *n* (%)	35 (32.4)	6 (6.1)	0.001
On cART, *n* (%)	108 (100.0)	102 (97.0)	0.222
cART Regime, n (%)			
Protease inhibitor-based therapy	34 (31.5)	11 (13.9)	0.001
Integrase inhibitor-based therapy	15 (13.9)	0 (0.0)	
NNRTI-based therapy	51 (47.2)	67 (84.8)	
Complex regime (> 1 of above)	8 (7.4)	1 (1.3)	
CD4 count, median (range), cells/mm^3^	640 (231–1243)	439 (41–1150)	0.001
Viral load, <40 copies/ml, n (%)	100(108)	*	
Comorbidities (more than 2), *n* (%)	71 (65.7)	27 (25.5)	0.001

* Viral load measurements not routinely available in clinical practice

#### Differences in musculoskeletal symptoms between the UK and Zambian groups

Musculoskeletal symptoms were common in both groups. However, no significant difference in the presence of any joint pain was observed (UK: 69% vs Zambia: 61%, p = 0.263). Whilst several comparisons were not statistically significant, the prevalence of symptoms was higher in the UK population compared to Zambia for all symptoms other than burning/gritty eyes. With regard to other symptoms, the UK group reported significantly more joint stiffness (UK: 50% vs Zambia: 40%), reduced joint movement (UK: 41% vs Zambia: 18%), muscle pain (UK: 57% vs Zambia: 38%), difficulty sleeping (UK: 52% vs Zambia: 24%) and fatigue (UK: 72% vs Zambia: 62%) compared to the Zambian group Table 2.

**Table 2:** Frequency of musculoskeletal symptoms.

*n* (%)	UK (*n* = 108)	Zambia (*n* = 106)	p-value
Joint pain	75 (69.4)	65 (61.2)	0.263
Muscle pain	61 (56.9)	40 (38.2)	0.012
Swollen/red joint	16 (14.7)	8 (7.8)	0.184
Reduction of joint movement	44 (40.8)	19 (18.0)	0.001
Joint stiffness	54 (50.0)	42 (39.9)	0.040
Difficulty in taking or gripping something	28 (25.5)	20 (18.6)	0.307
Burning/gritty eyes	24 (22.6)	25 (23.5)	1.000
Mouth sores	24 (22.4)	22 (20.8)	0.906
Skin rash	35 (32.4)	17 (15.8)	0.009
Nail changes	19 (17.9)	16 (14.7)	0.660
Dysuria	13 (12.3)	10 (9.9)	0.749
Difficulty sleeping	56 (52.3)	25 (23.5)	0.001
Fatigue	78 (72.0)	66 (61.8)	0.025

#### Differences in physical functioning and health-related quality of life

Despite the high frequency of musculoskeletal symptoms in both groups, all participants reported being able to perform most activities of daily living as assessed by the HAQ, with no significant differences in HAQ scores observed between the UK and Zambian groups (median HAQ score UK: 0 vs Zambia: 0, p = 0.749) Table 3. Although there were no differences in the frequency of symptoms and the capacity to perform activities of daily living between both groups, the UK group had significantly worse quality of life measurements compared to the Zambian group evidenced by lower scores on six SF-36 domains including physical functioning (UK: 90 vs Zambia: 95, p = 0.024), general health (UK: 62 vs Zambia: 75, p = 0.001), vitality (UK: 50 vs Zambia: 70, p = 0.001), social functioning (UK: 75 vs Zambia: 87.5, p = 0.018), emotional role (UK: 100 vs Zambia: 100, p = 0.003) and mental health (UK: 66 vs Zambia: 72, p = 0.001) Table 3.

**Table 3:** Stanford health assessment questionnaire (HAQ) and 36 short form survey health (SF-36) scores for the UK and Zambia groups.

Scores; median (IQR)	UK (*n* = 108)	Zambia (*n* = 106)	p-value
**HAQ**			
HAQ SDI*	0 (0–0.375)	0 (0–0.375)	0.749
HAQ ADI**	0 (0–0.375)	0 (0–0.250)	0.520
**SF-36 scale scores**			
Physical functioning	90 (65–100)	95 (75–100)	0.024
Physical role	100 (0–100)	100 (25–100)	0.499
Bodily pain	74 (42–100)	74 (52–100)	0.493
General health	62 (42–77)	75 (55–92)	0.001
Vitality	50 (30–70)	70 (50–80)	0.001
Social functioning	75 (50–100)	87.5 (62.5–100)	0.018
Emotional role	100 (0–100)	100 (66.67–100)	0.003
Mental health	66 (48–80)	72 (56–92)	0.001

* The Standard Disability Index

**The Alternative Disability Index; HAQ = (0 = no difficulty, 1 = some difficulty, 2 = much difficulty, to 3 = unable to do); SF-36 (0 = worst QoL; 100 = best QoL)

#### Associations between rheumatic symptoms, sociodemographic characteristics, and quality of life measurements in the UK group

The total variance explained by the multiple linear regression models for the UK group was 43.8%, 60.3% and 36.9% for the general health, vitality and mental health scores respectively. Table 4. The most important predictor of poorer general health, emotional, vitality and mental health was fatigue Table 4. Increasing joint or muscle pain severity was also associated with poorer general health and work status (unemployment) was associated with poorer mental health in the UK group.

**Table 4:** Summary of multiple regression analysis for variables predicting health scores for SF-36 domains in the UK group.

Variable	General health				Vitality				Mental health			
	B	β	p value	R^2^	B	β	p value	R^2^	B	β	p value	R^2^
				0.438				0.603				0.369
**(Constant)**	73.04		<0.001		91.73		<0.001		101.27		<0.001	
**Demographic factors**												
Age (years)	-0.34	-0.018	0.853		-0.029	-0.015	0.859		0.141	0.075	0.473	
Gender^1^	11.017	0.157	0.093		-2.699	-0.036	0.639		-9.147	-0.129	0.191	
Smoking^2^	-4.052	-0.089	0.347		-4.095	-0.085	0.284		-8.786	-0.191	0.059	
Comorbidities	-4.385	-0.098	0.285		2.949	0.062	0.417		-7.814	-0.173	0.077	
Work status^3^	-2.128	-0.049	0.624		-6.720	-0.146	0.084		-12.729	-0.288	0.007	
CD4 count^4^	0.003	0.030	0.727		-0.009	-0.090	0.222		-0.001	-0.006	0.949	
**Rheumatic symptoms**												
Reduced joint movement	1.460	0.034	0.728		-0.295	-0.006	0.937		-0.636	-0.015	0.887	
Joint or muscle stiffness	-5.930	-0.139	0.200		-1.267	-0.028	0.756		-1.687	-0.039	0.732	
Joint or muscle pain severity^5^	-1.952	-0.268	0.032		-0.424	-0.055	0.594		0.226	0.031	0.814	
Fatigue severity^5^	-2.484	-0.333	0.003		-5.229	-0.666	<0.001		-2.409	-0.320	0.006	

^1^Reference group is male

^2^Reference group is non-smoker vs being a current smoker

^3^Reference group is current employment vs current unemployment

^4^Unit of CD4 count is per 1 cell higher

^5^Unit of severity

#### Associations between musculoskeletal symptoms, sociodemographic characteristics, and quality of life measurements in the Zambian group

The total variance explained by the multiple linear regression models for the Zambia group was 23.2%, 28.7.3% and 21.2% for the general health, vitality and mental health scores respectively. Table 5. The only significant predictor of poorer general health, vitality and mental health was fatigue Table 5.

**Table 5:** Summary of multiple regression analysis for variables predicting health scores for SF-36 domains in the Zambia group.

Variable	General health				Vitality				Mental health			
	B	β	p value	R^2^	B	β	p value	R^2^	B	β	p value	R^2^
				0.232				0.287				0.212
**(Constant)**	69.12		<0.001		75.92		<0.001		53.245		<0.001	
**Demographic factors**												
Age (years)	-0.054	-0.033	0.803		0.146	0.088	0.481		0.290	0.170	0.198	
Gender^1^	-0.303	-0.008	-0.957		-3.227	-0.079	0.545		0.644	0.015	0.911	
Smoking^2^	14.14	0.157	0.212		6.010	0.066	0.578		7.227	0.077	0.535	
Comorbidities	-7.48	-0.162	0.226		-7.379	-0.158	0.213		-7.033	-0.146	0.270	
Work status^3^	7.49	0.185	0.149		-0.610	-0.015	0.901		4.863	0.116	0.362	
CD4 count^4^	0.009	0.119	0.385		0.006	0.073	0.573		0.016	0.200	0.145	
**Rheumatic symptoms**												
Reduced joint movement^5^	8.73	0.166	0.215		-2.689	-0.050	0.688		1.559	0.028	0.829	
Joint or muscle stiffness^5^	-2.19	-0.052	0.702		2.250	0.053	0.682		-4.800	-0.110	0.419	
Joint or muscle pain severity^5^	-0.83	-0.130	0.356		-1.163	-0.180	0.178		-0.568	-0.085	0.540	
Fatigue severity^5^	-2.04	-0.329	0.017		-2.608	-0.415	0.002		-1.906	-0.295	0.031	

^1^Reference group is male

^2^Reference group is non-smoker vs being a current smoker

^3^Reference group is current employment vs current unemployment

^4^Unit of CD4 count is per 1 cell higher

^5^Unit of severity

## Discussion

Despite effective cART, PLWH in both the UK and Zambia reported high rates of current musculoskeletal symptoms. Pain disorders have frequently been described in PLWH with a reported prevalence of 54% [32]. Our results are consistent with this estimate and go beyond by reporting the rate of other musculoskeletal symptoms in two different populations of PLWH. Musculoskeletal symptoms in PLWH can be difficult to interpret in clinical practice as overlapping symptoms from other pain disorders such as neuropathic pain, headaches (including a migraine), and other poorly defined pain syndromes are common [33]. Among the 214 patients included in our study, we identified fatigue, muscle and joint pain and joint stiffness as the most common self-reported symptoms. Participants from the UK reported higher frequency of almost all musculoskeletal symptoms. Possible explanations include average older age and longer disease duration, as shown by previous larger cohorts to have a significantly increased risk of developing rheumatic disease [34–36].

There is scanty information about the prevalence of these symptoms in the general population of Zambia. In the UK however, musculoskeletal conditions affect 1 in 4 of the adult population (many being young and of working age) which is around 9.6 million adults. The prevalence of regional and widespread musculoskeletal symptoms obtained from national surveys is estimated to be 28.7 per 100, rising with age to 36.6 per 100 among 70–79 years old with rates of disabling chronic pain of 14.3% in 18–25 years old and 62% in the over 75 age group [37, 38].

Among other musculoskeletal symptoms, fatigue was very prevalent in both groups with rates of 72% and 62% for the UK and the Zambian groups respectively. Interestingly, fatigue was the most common self-reported symptom and the only musculoskeletal symptom to predict poorer QoL across domains in both the UK and Zambia group. Various other studies agree with this finding, [35, 39, 40] suggesting fatigue has one of the greatest impacts on the QoL of PLWH [41]. Fatigue is a common symptom reported in individuals affected by other chronic musculoskeletal and inflammatory diseases such as systemic lupus erythematosus, seronegative and rheumatoid arthritis [42]. Whether fatigue in PLWH is influenced by the inflammatory effects of HIV, co-existing mental health disease or medications will be important to determine for future targeted interventions [35].

Although there were no significant differences in the impact of symptoms and the capacity to perform activities of daily living between both groups, the UK group had significantly worse quality of life measurements compared to the Zambian group evidenced by lower scores on six SF-36 domains including physical functioning, general health, vitality, social functioning, emotional role and mental health.

Perceptions on quality of life might vary by setting. A previous report looking at QoL measures across nine different resource-limited settings highlighted different cultural perceptions or variances in baseline characteristics beyond those we have data for, such as social support networks could account for differences in the QoL [43]. Access to specialist care, welfare, and medications to treat pain disorders differ significantly between the UK and Zambia, as a result individual resilience to pain and its impact on mental health and quality of life can be very different, which could explain some of our results.

Additionally, cultural and geographic differences in the way people respond to or understand the questionnaires may account for some of these differences. The cross-cultural use and adapated versions have however proven equally reliable and valid as their parent [23]. Although our study was not powered to validate these questionnaires, with the help of an interpreter items were culturally-adapted and differences in understanding was minimised.

Many reports suggest increasing age, the presence of comorbidities and polypharmacy are associated with a poorer QoL for PLWH [35]. We found unemployment was associated with the worse mental health in the UK but age and presence of co-morbidities were not associated with quality of life domains in Brighton or Zambia.

It is surprising that there is such a big difference in the proportion of participants reporting >2 comorbidities in the two countries, particularly given the lower CD4 count among the Zambian patients. This may reflect a difference in documentation and the feasibility to diagnose multiple conditions between the two populations rather than a true difference in number.

Our study has several important limitations. Firstly, the cross-sectional study design means we are unable to conclude temporality in the associations found and should, therefore, be interpreted with caution. Secondly, our relatively small sample size of 214 participants (108: UK; 106: Zambia) means we may have made a type 2 error. Given the small size of the study only some of the larger comparisons are statistically significant. Had our sample size been larger our results may have been different. For example, although there was no significant difference in the prevalence of joint pain between the two populations, the overall prevalence difference of 8% could in fact be a true difference. It is important to highlight that our study was not powered to detect clinically significant differences between Brighton and Lusaka and was rather exploratory in nature. In addition, the possibility of type I errors should also be considered. Thirdly, participants were recruited by a healthcare professional through convenience sampling. Although this is regularly used in studies related to HIV due to ongoing issues of stigma, this may have increased the potential for selection bias. Additionally, although our clinics are large and demographically representable of PLWH in Brighton and Lusaka, they are only single centres and as such, limit the generalisability of our results to the local environment rather than other institutions. Other limitations include the absence of a detailed description of the characteristics of the pain these patients experienced, social support networks available to them and not adjusting for other potentially important confounders, including longer disease duration, which is known to be associated with increased rates of musculoskeletal symptoms.

On the other hand, this study is unique in its attempt to compare musculoskeletal symptom prevalence and QoL impact in PLWH from a high and lower resource setting. Highlighting variability in associations in different settings and areas that require attention to ensure the best QoL for PLWH.

The mechanisms by which HIV may lead to an increased risk of pain syndromes remains unclear, but it is likely to be multifactorial, involving biological (inflammation), psychological and social factors [11]. Considering the high prevalence of pain syndromes in PLWH and their impact on mental health, functional status and quality of life designing optimal and cost-effective pathways for diagnosis and management of pain should be a priority of HIV care regardless of the geographical setting.

The public health impact of musculoskeletal pain in PLWH cannot be underestimated. Individuals with musculoskeletal pain are less likely to be in work than those without a health problem and are also more likely to retire early. The direct cost to the economy on days lost of work can be substantial as those affected by pain syndromes are more likely to utilize healthcare services and require drug prescriptions to control the symptoms [44]. Longitudinal studies in high- and low-income settings investigating the contributors of musculoskeletal pain are needed to inform the development of effective interventions to prevent and managed those with musculoskeletal symptoms living with HIV.

## Conclusions

In conclusion, our findings indicate that the proportion of PLWH that are affected by musculoskeletal symptoms remains high, independent of the clinical setting. We have also demonstrated a complex interplay between sociodemographic and physical symptoms on the impact of health-related QoL.

## Supporting information

S1 File Sociodemographic questionnaire in Bemba. (PDF)

S2 File Rheumatological symptoms questionnaire in Bemba. (PDF)

S3 File HAQ in Bemba. (PDF)

S4 File SF-36 questionnaire in Bemba. (PDF)

S5 File Sociodemographic questionnaire in Nyanja. (PDF)

S6 File Rheumatological symptoms questionnaire in Nyanja. (PDF)

S7 File HAQ in Nyanja. (PDF)

S8 File SF-36 in Nyanja. (PDF)

S9 File Rheumatological symptoms questionnaire in English. (PDF)

S10 File Sociodemographic questionnaire in English. (PDF)
