# Supplementary material for: Impact of musculoskeletal symptoms on physical functioning and quality of life among treated people with HIV in high and low resource settings: A case study of the UK and Zambia
Source: PLoS One. 2019 May 13;14(5):e0216787. doi: 10.1371/journal.pone.0216787 (PMC6513081; doi:10.1371/journal.pone.0216787)
Supplement: S1 File — (PDF) [file pone.0216787.s001.pdf]

Socio-demographic data:

Patient Number:

Umwaume ☐ Umwanakashi ☐

imyaka ☐ Imyaka panshita yakusangwa nobulwele ☐

Imyaka inga elyo tamulasanga nobulwele ☐

Apapelela amasambililo: Takuli ☐ Isukulu lyakwambilapo ☐

Isukulu likalamba (sekondali) ☐ Isukulu likalamba ilya koleji ☐

Isukulu likalamba ilya yuniveziti ☐

Umupelela ukufola (pamwaka): ≤ ukulingana na K10 000 nangula panshi ☐

K10 000 ukufika ku 30 000 ☐ K30 000 ukufika ku 50 000 ☐ K50 000 nokucilapo ☐

Icupo: Ukupwa ☐ Ukukana upwa ☐ ukwenda naumo ☐

Ukubomba: ubanincito ☐ Ukukanabomba ☐

Incito:

Bushe nimwe mufola mulupwa: Emukwai ☐ Iyo ☐

Icicetakelo:

Balapepa fwaka: Emukwai ☐ Iyoo ☐

BMI (Ubutali nokufina):

Inshila yakwambwilamo: Abaume nabanakashi ☐ abaume/abanakashi bekabeka ☐

mumulopa ☐ Ukufuma kulinyina ukuya kumwana ☐

Co-morbidities (bushe mwalikwata amalwele yambi ayakunwa umuti (medical), ikuputulwa (surgical) nangula ayakumutima/amatontonkanyo (psychological)

Ayakunwa umuti	
Ayakuputulwa	
Ayamumutima/amatontonkanyo	

Bushe mwalikwata ?

Hepatitis B: Emukwai ☐ Awe ☐

Hepatitis C: Emukwai ☐ Awe ☐ Ukupola bwino bwino ☐

Tuberculosis: Emukwai ☐ Awe ☐ Ukupola bwino bwino ☐

Amalwele yambi ayakusenda mukulalana: Emukwai ☐ Awe ☐ Ukupola bwino bwino ☐

Ngacakutila cilifi, yesa? Mukwai peleni ubulondoloshi panshi pamo nga. Ukusangwa, nokucecentwa:

Imiku mwatekwa mucipatala panuma ya kusangwa nobulwele: ☐

Mukwai peleni ubulondoloshi pali cila kutekwa pamo nga Ukusangwa, nokucecentwa

Impenwa ya CD4 count iyakulekelesha: ☐

Impenwa ya viral load iyakulekelesha: ☐

Bushe mulenwa ama ARV?   Emukwai ☐   Awe ☐

Mutundu nshi wa muti wa ma ARV eyo munwa ?

Mukwai peleni ubulondoloshi pamo nga inshiku, impendwa yamuti munwa:
