# Supplementary material for: Impact of musculoskeletal symptoms on physical functioning and quality of life among treated people with HIV in high and low resource settings: A case study of the UK and Zambia
Source: PLoS One. 2019 May 13;14(5):e0216787. doi: 10.1371/journal.pone.0216787 (PMC6513081; doi:10.1371/journal.pone.0216787)
Supplement: S2 File — (PDF) [file pone.0216787.s002.pdf]

Amepusho:

Ufilangililo fya bulwele bwabukali bwa mumubili:

Amepusho yalekonka yalelola kufintu limbi ifyacitike ukufuma epo mwaishiba ukuti mwalikwata HIV. Mukwai congeni mukabokoshi ngefilefwaika.

Bushe mulakwata ubukali mufiputulwa fyamumubili? Emukwai ☐ Awe ☐

Ngacili ngefi langeni amaka yabukali ukubomfya sikelo uuli panshi?

Ukukana umfwa ubukali	0	1	2	3	4	5	6	7	8	9	10	Ukukalipa sana

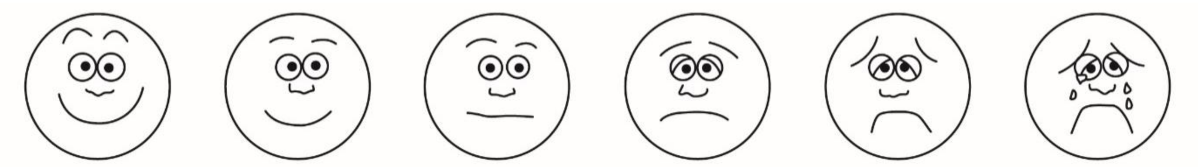

Ngacili ngefi, mukwai lembeni pafimpashanya ifili panshi, incende emo mumfwa ubukali?

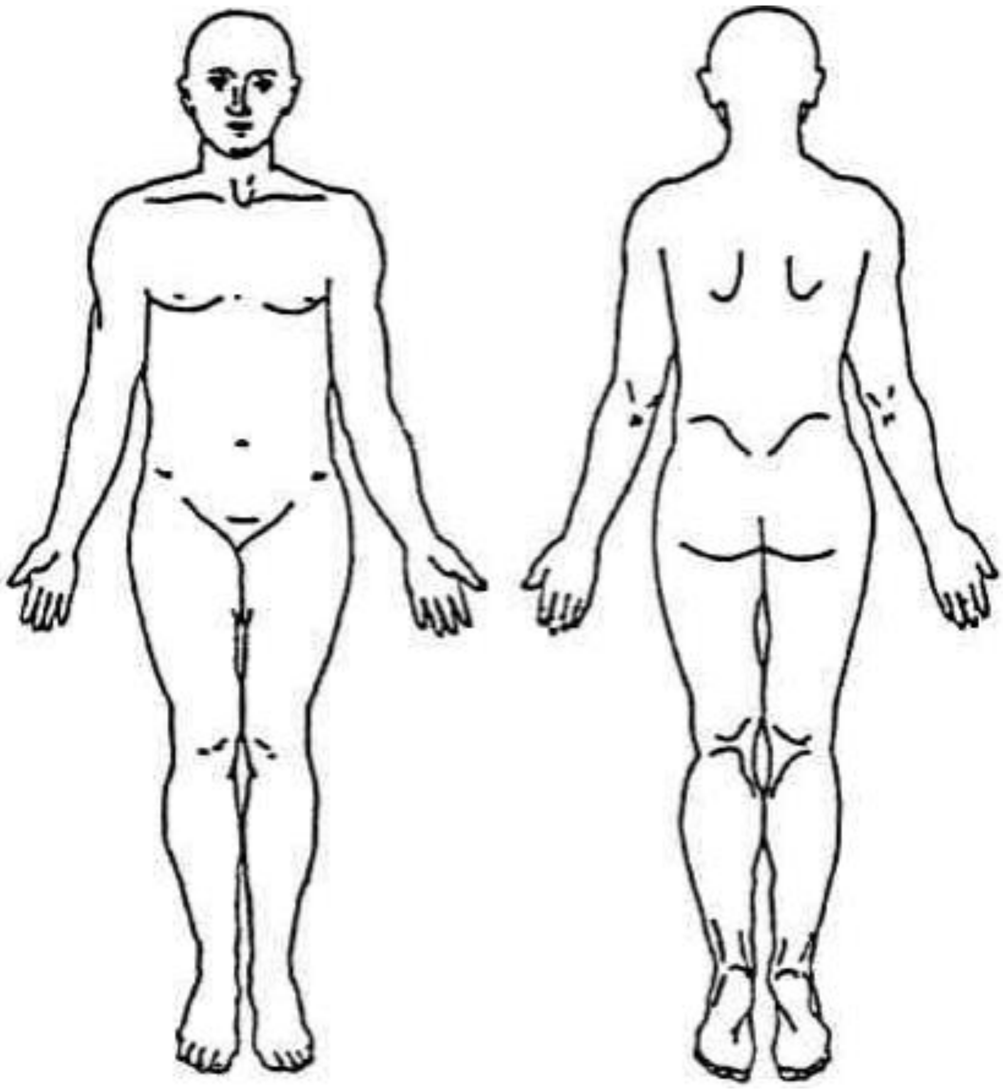

Bushe mwalibala amufimbapo mufiputulwa fya mumubili? Emukwai ☐ Awe ☐

Bushe mwalubala amumonapo ukubwekela panshi ukwa kusela kwa mufiputulwa fya mumubili?

Emukwai ☐ Awe ☐

Bushe mulakwata ukukosa kwa mufiputulwa fyamumubili nangula mumunofu ulucelo?

Emukwai ☐ Awe ☐

(ukukosa tulepilibula inshita kuba ubwafya ukusesha umunofu nokukontola ifiputulwa fyamubili; nafikosa)

Bushe ninshita yalepa shani ukukosa kusenda ukufuma inshita yakubuka?

Panshi ya insa amakumi yatatu ☐ Ukucila insa amakumi yatatu ☐

Bushe mwalibala amukwatapo ubukali mumunofu? Emukwai ☐ Awe ☐

Ngacili ngefi langeni amaka yabukali ukubomfya sikelo uuli panshi?

Ukukana umfwa ubukali	0	1	2	3	4	5	6	7	8	9	10	Ukukalipa sana

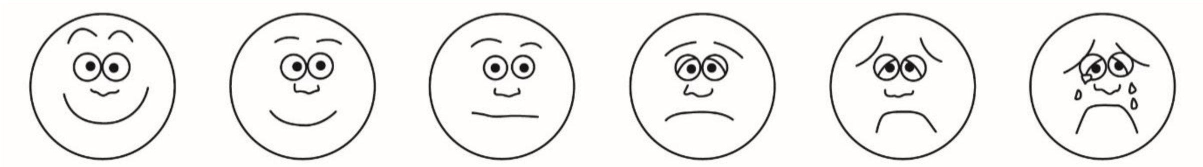

Bushe mwalibala amukwata ubukali nokukosa kwamukosi? Emukwai ☐ Awe ☐

Bushe mwalibala amukwata ubukali nokukosa kwamukosi? Emukwai ☐ Awe ☐

Bushe mwalibala amumonapo ubukali ubulibonse mututende? Emukwai ☐ Awe ☐

Bushe mulakwakapo ubwafya pakwikata/ukufina icintu? Emukwai ☐ Awe ☐

Bushe amenso yenu yalakaba inshita yonse, ukumfwika ukuma nangula ngumuli amabwe?

Emukwai ☐ Awe ☐

Bushe mwalikwatapo ifilonda mukanwa? Emukwai ☐ Awe ☐

Bushe mumaboko mwenu mulabuta nangula ukufitilila ngamwaikata ifyatalala?

Emukwai ☐ Awe ☐

Bushe mwalikwatapo utulonda pamubili? Emukwai ☐ Awe ☐

Ngacili ngefi, londololeni ifyo utulonda twali?

Bushe mwalimona ubupusano mumaala yenu? Emukwai Awe

Bushe mwalimonapo ukukana umfwa bwino pakuposa amenshi? Emukwai Awe

Bushe mwalikwatapo ubwafya pakulala? Emukwai Awe

Bushe mwalyumfwapo ukunaka sana? Emukwai Awe

Ngacili ngefi langeni amaka yakunaka ukubomfya sikelo uuli panshi?

Ukukana naka	0	1	2	3	4	5	6	7	8	9	10	Ukunaka sana

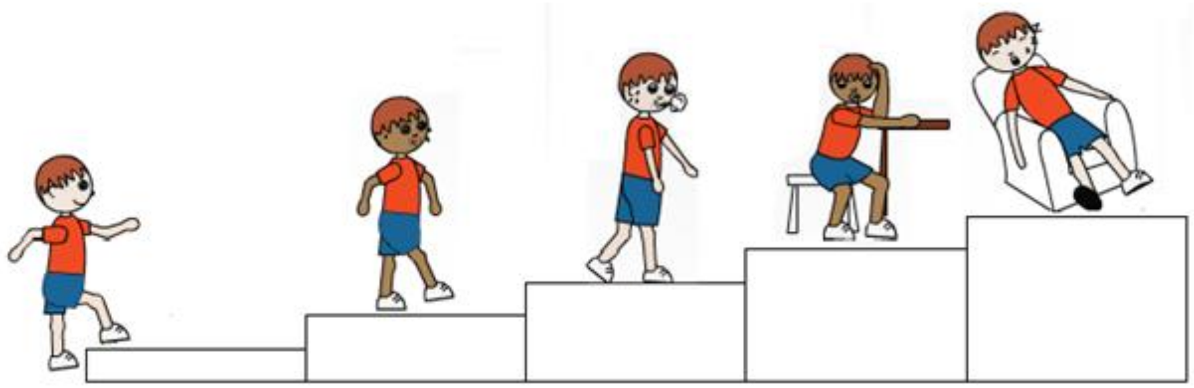

Bushe mwiliumfwapo ukunenuka? Emukwai Awe

Bushe mwalumonanapo naba Rheumatologist (ba shinganga balolekesha pamalwele ya mumunofu namufiputulwa fya mumubili)?

Emukwai Awe

Bushe mwalilwalapo ubulwele lwa mumunofu namufiputulwa fya mumubili na mumishipa?

Emukwai ☐ Awe ☐

Nga cilifi, kufwailishanshi kwacitike? Mukwai londololeni pamu nga ukupima umulopa, mucifuba:

Bushe kuli umo mulupwa lwenu uwalwalapo ubulwele lwa mumunofu namufiputulwa fya mumubili na mumishipa?

Emukwai ☐ Awe ☐

Ngacilifi, ninani elyo bulwele nshi?
