# Supplementary material for: Impact of musculoskeletal symptoms on physical functioning and quality of life among treated people with HIV in high and low resource settings: A case study of the UK and Zambia
Source: PLoS One. 2019 May 13;14(5):e0216787. doi: 10.1371/journal.pone.0216787 (PMC6513081; doi:10.1371/journal.pone.0216787)
Supplement: S3 File — (PDF) [file pone.0216787.s003.pdf]

Ilya mepusho:

Ukupima Ukukwanisha Kwamilimo Ukubomfya Icipepala Cakufwailikisha Palwa pa Bumi

Mukwai congeni ubwasuko ubulelondelola bwino efyo ukukwanisha kwenu ukwapali cila bushiku mu mulungu uyu uwapita

	Ukuwabula ubwafya	Nobwafya ubunono	Nobwafya sana	Ukukana kwanisha
**1. Ukufwala nokuiwamya**				
Bushe mulakwanisha:				
a. Ukufwala mwebene, ukulundapo nokukaka insapato nukukaka ama batani?	☐	☐	☐	☐
b. Ikuwamya/ukusamba mumishishi?	☐	☐	☐	☐
**2. Ukwima**				
Bushe mulakwanisha:				
a. Ukwima pacipuna icabula ifyakwikatilako?	☐	☐	☐	☐
b. Ikwingila nokufuma mubusanshi?	☐	☐	☐	☐
**3. Ukulya**				
Bushe mulakwanisha:				
a. Ukuiputwila inama?	☐	☐	☐	☐
b. Ikwimya inkomoki nangula itambula ilyaisula ukufika kukanwa?	☐	☐	☐	☐
c. Ukwisula icibokoshi ca mukaka icipya (nangula isopo lya bunga ilyakucapila) ?	☐	☐	☐	☐

	Ukuwabula ubwafya	Nobwafya ubunono	Nobwafya sana	Ukukana kwanisha
**4. Ukwenda**				
Bushe mulakwanisha Are:				
a. Ukwenda panse pamushili uwalingana?	☐	☐	☐	☐
b. Ukunina pamulu apali impendwa iyapakunina ukuyapamulu isano?	☐	☐	☐	☐
**5. Ubusaka**				
Bushe mulakwanisha :				
a. Ukusamba nokupukuta umubili wenu onse?	☐	☐	☐	☐
b. Ukusamba?	☐	☐	☐	☐
c. Ukunina nokufuma pa cimbusu?	☐	☐	☐	☐
**6. Ukufika**				
Bushe kuti mwakwanisha:				
a. Ukwimya nokutula panshi icintu cafina impendwa shisanu -5 lb (icapala isaka lya imambati) ukufuma pancende iicilile pamutwe panono?	☐	☐	☐	☐
b. Ukukontama nokusenda ifyakufwala ukufuma panshi?	☐	☐	☐	☐
**7. Ukufina**				
Bushe mulakwanisha:				
a. Ukwisula ifibi fya myotoka?	☐	☐	☐	☐
b. Ukwisula amabotolo ayaisulwapo kale?	☐	☐	☐	☐
c. Ukwisula nokwisala pompi wamenshi	☐	☐	☐	☐

8. Ifyakucita	Ukuwabula ubwafya	Nobwafya ubunono	Nobwafya sana	Ukukana kwanisha
Bushe mulakwanisha:				
a. Ukubomba imilimo yacila bushiku iya pang'anda nokushita?	☐	☐	☐	☐
b. Ukwingila nokufuma muli ba motoka?	☐	☐	☐	☐
c. Ukubomba imilimo ya mung'ganda pamo nga ukufumya ulukungu naba mashini, imilimo ya mug'ganda elyo nokubomba mumabala ukwayanguka?	☐	☐	☐	☐

Mukwai congeni ifyakwafwilisha nangula ifibombelo efyo mubomfya ilingi mumilimo yonse iyi:

Inkonto ☐

Inkonto yakwendamo ☐

Ifyakubomfya ifya ibela ifyakubumba nangula ifyaibela ☐

Inkonto yakwendela ☐

Icipuna cakwendelamo ☐

icipuna ickwendelamo icaibela icakupanga ☐

Icimbusu cabela pamulu ☐

Icipuna cakusambilapo ☐

Icakushintilishapo pakusambal ☐

Ifyakusendelako ifili apatali ☐

Icakwiswilako amabotolo (ica mabotolo yaisulwa kale) ☐

Ifyakubomfya pakufwala (amabatani, zipu, insapato) ☐

Fimbi (Mukwai lonololeni): Takuli ☐

Mukwai congeni ifilifyonse mufwayamo ubwafwilisho ukufuma kulibambi ilingi:

Ukufwala nokuiwamya ☐

Ukulya ☐

Ukwima ☐

Ukwenda ☐

Ubusaka ☐

Ukufina nokwisula ifintu ☐

UkufikaReach ☐

Imilimo sha lyonse elyo neshapang'anda ☐

Takuli ☐
