# Supplementary material for: Impact of musculoskeletal symptoms on physical functioning and quality of life among treated people with HIV in high and low resource settings: A case study of the UK and Zambia
Source: PLoS One. 2019 May 13;14(5):e0216787. doi: 10.1371/journal.pone.0216787 (PMC6513081; doi:10.1371/journal.pone.0216787)
Supplement: S4 File — (PDF) [file pone.0216787.s004.pdf]

Amepusho:

SF- 36 Questionnaire – Imibele ya bumi

Mukwia congeni ubwasuko ubulelondolola bwino fintu mukwanisha ifya cila bushiku elyo napalwabumi

Ubumi bonse

1. ukulolekesha fyonse, kuti mwalanda ukuti muleyumfwa shani ubumi?

Ubusuma ukupitilila	☐	Ubusuma sana	☐	Ubusuma	☐	Efilyako	☐	Ububi	☐

2. Ukulinganya nomwaka wafumapo, kuti mwalanda ukuti muleyumfwa shani ubumi mukulolekesha fyonse nomba?

Kwawamapo ukucila umwaka wafumapo	☐
Kwawamapo panono ukucila umwaka uwapita	☐
Ukulinganako	☐
Ukubipapo panonon ukucila umwaka wapiti	☐
Ububi sana ukucila umwaka wapiti	☐

Ukupelelwa kwa mumilimo

Ifintu fililekonka fili pamilimo mungabomba mubushiku ubuli ngenshiku shonse. Bushe ubumi bwenu bulemikanya nomba mumilimo ishi? Ngacilifi munshilanshi?

3. Incite shakosa, pamo ngokubutuka, ukwimya ifyafina, ikusenda ulubalii mu mangalo ayakuso.

Emukwai, ndefilwa sana	Emukwai ndefilwa panono	Awe, nshilefilwa napanono

4. Imilimo yapakati pamo nga, ukusesha itebulo, mashini yakufumya ulukungu, ukuteya amangalo yakuposa umupila nokuma nenkonto. (bowling nangula golf).

Emukwai, ndefilwa sana	Emukwai ndefilwa panono	Awe, nshilefilwa napanono

5. Ukwimya ifyakushita

Emukwai, ndefilwa sana	Emukwai ndefilwa panono	Awe, nshilefilwa napanono

6. Ukunina imitantu iyingi iya mucikulwa

Emukwai, ndefilwa sana	Emukwai ndefilwa panono	Awe, nshilefilwa napanono

7. Ukunina umutanto umouwamucikulwa

Emukwai, ndefilwa sana	Emukwai ndefilwa panono	Awe, nshilefilwa napanono

8. Ukwinama, ukufukama nangula ukukontama

Emukwai, ndefilwa sana	Emukwai ndefilwa panono	Awe, nshilefilwa napanono

9. Ukwenda ubutali ubulecila imailo limo

Emukwai, ndefilwa sana	Emukwai ndefilwa panono	Awe, nshilefilwa napanono

10. Ukenda panshi ukupitilila ifikulwa ifingi

Emukwai, ndefilwa sana	Emukwai ndefilwa panono	Awe, nshilefilwa napanono

11. Ukwenda panshi ukupita icikulwa cimo

Emukwai, ndefilwa sana	Emukwai ndefilwa panono	Awe, nshilefilwa napanono

12. Ukusamba nangula ukufwala mwebene

Emukwai, ndefilwa sana	Emukwai ndefilwa panono	Awe, nshilefilwa napanono

Amafya ya ku mubili

Mumilungu yapita ine bushe mwalikwatako amafya ayalekonkapo kunchito yenu nangula kumilimo ya cila bushiku umulandu wa mibele ya bumi kumubili?

13. Ukucefyako inshita mwasendele kuncito nangula imilimo imbi

Emukwai	Awe

14. Ukukana fika ngepo mungafwaya

Emukwai	Awe

15. Mwalifilwa mumibele ya ncito nangula imilimo imbi.

Emukwai Awe

16. Mwalisanga ubwafya ukubomba incito nangula imilimo shimbi (ukupalanya, incito yalikosesha)

Emukwai Awe

Amafya yabulwele wa matontonkanyo

Mumilungu ine iyapita, bushe mwalikwatako amafya ayalekonkapo kunchito nangula ku milimo imbi pamulandu wamafya yamatontonkanyo (pamo nga ukumfwa icikonko nangula ukucetekela) During the past 4 weeks, have you had any of the following problems with your work or other regular daily activities as a result of any emotional problems (such as feeling depressed or umwenso)?

17. Ukucefyako inshita mwasendele kuncito nangula imilimo imbi

Emukwai Awe

18. Ukukana fika ngepo mungafwaya

Emukwai Awe

19. Tamwabombele incite nangula imilimo imbi nobulondo ngalyonse

Emukwai Awe

Imilimo yakwampana

20. Mumilungu ine iyapita, nimunshila nshi umo amafya ya kumumubili namumatontonkanyo yalenga ukukana ba bwino mukwampana kwenu nabalupwa, ifibusa, abena mupalamano nangula utubungwe?

Takuli panono Pakati Sana Icabipa sana

Ubukali

21. Bukali bwakosa shani ubwamumubili ebo mwakwete mumilungu ine iyapita?

Takuli nangula panono Panono sana panono pakati Sana Ikubipa sana

22. Mumilungu ine iyapita, nimunshila nshi ubukali bwaonawile incito yenu iyalyonse (ikulundapo incito shapanse yang'anda neyamung'anda)?

Takuli nangula panono	panono	pakati	mubwingiko	Sana

Amaka elyo namatontonkanyo

Aya amepusho yapafyo muleumfwa elyo nefyo ifintu fyali kuli imwe mumilungu ine iyapita. Palipusho lyonse, mukwai peleni ubwasuko ubupalamine nefyo mwaleumfwa.

23. Bushe muliyumfwa abansansa (ukumfya amaka nokutemwa sana)?

Yonse inshita	Ubwingi bwa nshita	Ukupalama ubwingi bwanshita	Inshita shimo shimo	Munshita ishinono	Taficitika inshita iiliyonse

24. Bushe mwalibapo umuntu wamwenso sana?

Yonse inshita	Ubwingi bwa nshita	Ukupalama ubwingi bwanshita	Inshita shimo shimo	Munshita ishinono	Taficitika inshita iiliyonse

25. Bushe mwalibapo ababula insansa icakuti tapali nefinga lenga ukumisekeshako?

Yonse inshita	Ubwingi bwa nshita	Ukupalama ubwingi bwanshita	Inshita shimo shimo	Munshita ishinono	Taficitika inshita iiliyonse

26. Bushe mulomfwa umutimfa ukutalala elyo nomutende?

Yonse inshita	Ubwingi bwa nshita	Ukupalama ubwingi bwanshita	Inshita shimo shimo	Munshita ishinono	Taficitika inshita iiliyonse

27. Bushe mwalikwata amaka ayengi?

Yonse inshita	Ubwingi bwa nshita	Ukupalama ubwingi bwanshita	Inshita shimo shimo	Munshita ishinono	Taficitika inshita iiliyonse

28. Bushe mwaliyumfwapo abacikonko kumutima elyo nokukanaba nensansa?

Yonse inshita	Ubwingi bwa nshita	Ukupalama ubwingi bwanshita	Inshita shimo shimo	Munshita ishinono	Taficitika inshita iiliyonse

29. Bushe mwaliyumfwapo ukunaka sana?

Yonse inshita	Ubwingi bwa nshita	Ukupalama ubwingi bwanshita	Inshita shimo shimo	Munshita ishinono	Taficitika inshita iiliyonse

30. Bushe mwalibapo umuntu wansansa?

Yonse inshita	Ubwingi bwa nshita	Ukupalama ubwingi bwanshita	Inshita shimo shimo	Munshita ishinono	Taficitika inshita iiliyonse

31. Bushe mwaliumfwa ukunaka?

Yonse inshita	Ubwingi bwa nshita	Ukupalama ubwingi bwanshita	Inshita shimo shimo	Munshita ishinono	Taficitika inshita iiliyonse

Imilimo shakwampana

32. Mumilungu ine iyapita, ninshita iyinga shani amafya ya kumumubili namumatontonkanyo yalenga ukukana ba bwino mumilimo ya kwampana kwenu (pamo ngo kutandalila ifibusa, balupwa, nabambi)?

Yonse inshita	Ubwingi bwa nshita	Ukupalama ubwingi bwanshita	Inshita shimo shimo	Munshita ishinono

Ukulolekesha palwabumi konse konse

Bushe muli ifishinka nangula iyoo mumashiwi ayasoswa kulubali lwenu?

33. Mboneka ukuba uwakwangufyana ukulwalilila ukucila abantu bambi

Cishinka icabula nokutwishika	Cishinka ilingiline	Nshishibe	Tefyo ilingi	Tefyo icabula nokutwishika

34. Ndimutuntulu ngomuntu uulu oonse naishiba

Cishinka icabula nokutwishika	Cishinka ilingiline	Nshishibe	Tefyo ilingi	Tefyo icabula nokutwishika

35. Ndemona ubumi bwandi ukuba ubwabipa

Cishinka icabula nokutwishika	Cishinka ilingiline	Nshishibe	Tefyo ilingi	Tefyo icabula nokutwishika

36. Ubumi bwandi buli bwino sana

Cishinka icabula nokutwishika	Cishinka ilingiline	Nshishibe	Tefyo ilingi	Tefyo icabula nokutwishika
