# Supplementary material for: Impact of musculoskeletal symptoms on physical functioning and quality of life among treated people with HIV in high and low resource settings: A case study of the UK and Zambia
Source: PLoS One. 2019 May 13;14(5):e0216787. doi: 10.1371/journal.pone.0216787 (PMC6513081; doi:10.1371/journal.pone.0216787)
Supplement: S5 File — (PDF) [file pone.0216787.s005.pdf]

Socio-demographic data:

nambala la odwala:

Amuna ☐ Akazi ☐

Zaka ☐ Zaka pomwe mu na onedwa ☐ Ndi zaka zingati mukalibe ku onedwa ☐

None ☐ Primary ☐ Secondary ☐ College ☐ University ☐

Muyeso wa ndalama zomwe mulandila: ≤ K10 000 ☐ K10 000 – 30 000 ☐

K30 000 – 50 000 ☐ ≥ K50 000 ☐

Za Chikwati: Ndinu okwatila/kwatila ☐ osakwatila ☐ kusewenzela pamodzi ☐

Kumbali Za Nchito: Munthu olembedwa nchito ☐ Amene Sasewenza ☐

Nchito:

Kodi ndi mwe mu sewenza ba banja lanu: Ndi bvomekeza ☐ Sindi bvomekeza ☐

Ndi wa chipembezo:

Ndi bvomekeza ☐ Ndi bvomekeza ☐

kupima (utali Ndi Kulema):

Njila zo tengelamo kadoyo : ku gonana ndi amuna/akazi ☐ ku khuza zinthu za magazi ☐

Amai kupsila mwana kadoyo ☐

Kodi muli ndi mabvuto monga aya?

Zolinga ku Mankwala ndi chipatala	
Zo tumbula	
Zolinga ku Maganizo	

kodi muli ndi?

Hepatitis B: Ndi bvomekeza ☐ Sindi bvomekeza ☐

Hepatitis C: Ndi bvomekeza ☐ Sindi bvomekeza ☐ Ana polesedwa bwino ☐

Matenda a TB: Ndi bvomekeza ☐ Sindi bvomekeza ☐ Ana polesedwa bwino ☐

Matenda ena akatengela: Ndi bvomekeza ☐ Sindi bvomekeza ☐ Ana polesedwa ☐

Ngati ndi telo ndi liti? onetsani pansi apa

Unyinji wa mphamvu za mthupi: Ndi bvomekeza ☐ Sindi bvomekeza ☐

Unyinji wa kadoyo ka HIV:

Ndi bvomekeza ☐ Sindi bvomekeza ☐

Kodi mukumwa mankwala a ma ARVs?

Ndi ma ARVs otani a mene mu kumwa?

Tikupemphani kuti muti udzeko za pa tsiku ndi za ka mwedwe ka mankwala kuti ndi angati?

