# Supplementary material for: Impact of musculoskeletal symptoms on physical functioning and quality of life among treated people with HIV in high and low resource settings: A case study of the UK and Zambia
Source: PLoS One. 2019 May 13;14(5):e0216787. doi: 10.1371/journal.pone.0216787 (PMC6513081; doi:10.1371/journal.pone.0216787)
Supplement: S6 File — (PDF) [file pone.0216787.s006.pdf]

Pepala la ma funso:

Zisonyezo Za Ku Bvimba Mu malungo:

Ma funso osatilapo alinga ku zinthu zi mene zina chitika ku chokela pa mene mu na dziwa kuti muli ndi ka doyo ka HIV. onetse tsani kuti mu chonga pa malo oyenera.

Kodi mu na nkalako ndi zo wawa mu malungo? Ndi bvomekeza ☐ Sindi bvomekeza ☐

ngati nitelo, kodi mungati langize muyeso mwa ku sewenzesa mpimo wa zo wawa uyu

No Pain	0	1	2	3	4	5	6	7	8	9	10	Worst pain

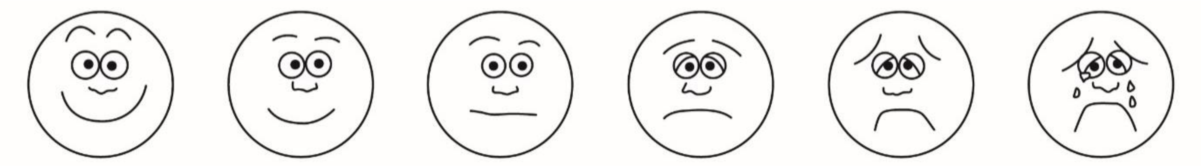

Ngati ntelo, onetsani mwa ku lemba pa ma thupi la anthu awili awa, pa malo amene mukumva kuwawa?

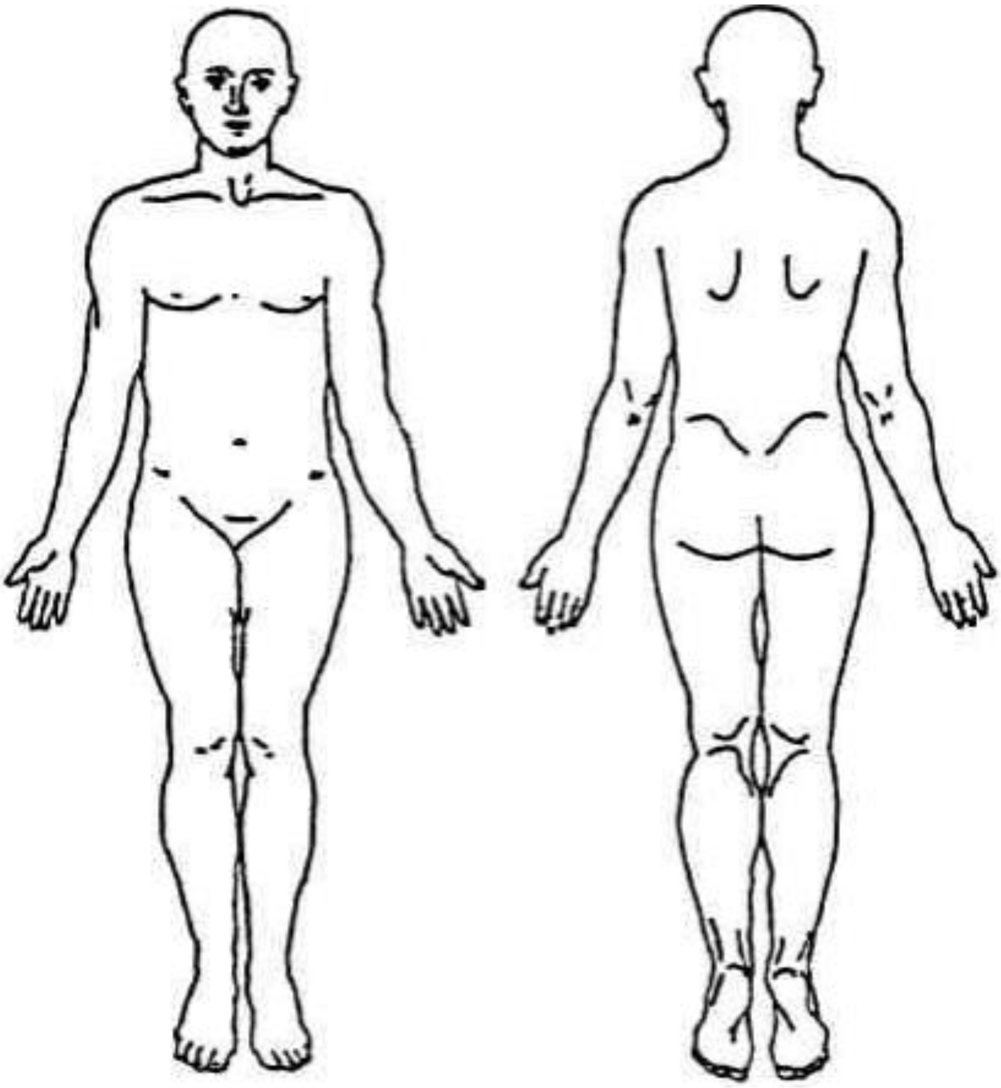

Nanga munkalapo obvimba ndi kusweta muma joini?

Ndi bvomekeza ☐ sindi bvomekeza ☐

Kodi muna mnvelako ku chepekela kwa ku nyanganya kwa ma malungo?

Ndi Bvomekeza ☐ sindi bvomekeza ☐

Kodi mu ma nkalako ndi ku kosa mu malungo kapena mphamvu mawa mawa?

Ndi bvomekeza ☐ sindi bvomekeza ☐

kodi ndi nthawi la itali bwanji/lo tani la mene mu ma mvela ku kosa kwa kosi kuchokela pa mene mu ma ukila?

ku chepekela mphindi makumi atatu ☐ Kupitilila mphindi makumi atatu ☐

kodi mu na nkalako ndi zo wawa mu mphamvu za mthupi?

Ndi bvomekeza ☐ sindi bvomekeza ☐

Ngati ni mwa mene, langizani ukali wake mwa ku sewenzesa mpimo/muyeso uyu ulipansi?

No Pain	0	1	2	3	4	5	6	7	8	9	10	Worst pain

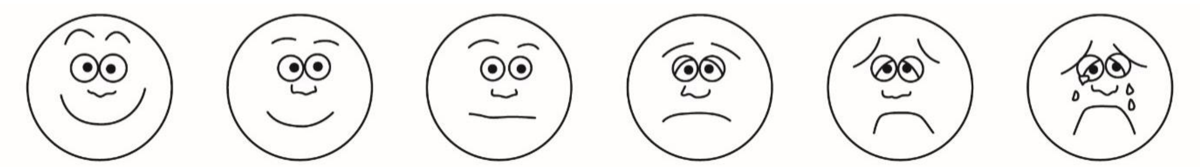

kodi muna nkalako ndi ku wawa kuli konse kwa kosi lanu kapena kosi ku kosa?

Ndi bvomekeza ☐ Sindi bvomekeza ☐

kodi mwa nkalako ndi ku wawa kapena ku kosa kwa msana?

Ndi bvomekeza ☐ Sindi bvomekeza ☐

Nanga mwa mvelako kuwawa ku ka denene kanu? kunsi kwa mendo, ko dyakila, ku ka skon

Ndi bvomekeza ☐ Sindi Bvomekeza ☐

kodi mu na nkalako ndi mabvuto pa ka tengedwe kapena mu ma gwilidwe kapena mu ka finidwe ka zinthu?

Ndi bvomekeza ☐ Sindi bvomekeza ☐

kodi menso anu ama pitilizabe kupya, kuyuma, kapena ku mvela ngati muli myala?

Ndi bvomekeza ☐ Sindi bvomekeza ☐

kodi mu na nkalapo ndi zilonda mu kamwa mwanu?

Ndi bvomekeza ☐ Sindi bvomekeza ☐

Kodi manja anu a ma sintha ku nkala yo yera kapena ku biliwila ngati kwa zizila?

Ndi bvomekeza ☐ Sindi bvomekeza ☐

Kodi muna nkhalapo ndi to tupa pa thupi?

Ndi bvomekeza ☐ Sindi bvomekeza ☐

Ngati nitelo ndi to tupa totani?

kodi mu na onako kusintha kuli konse mu njala zanu

Ndi bvomekeza ☐ Sindi bvomekeza ☐

kodi simuna mveko zowawa zili zonse pa ku taya madzi?

Ndi bvomekeza ☐ Sindi bvomekeza ☐

kodi mwa nkalako obvutikila pa ku gona?

Ndi bvomekeza ☐ Sindi bvomekeza ☐

Kodi mwa mvelako kulema?

Ndi Bvomekeza ☐ Sindi bvomekeza ☐

Ngati ntelo langizani kulema kwake mwa mwa ku sewenzesa mpimo uyu uli apa pansi?

Not Fatigued	0	1	2	3	4	5	6	7	8	9	10	Extremely fatigued

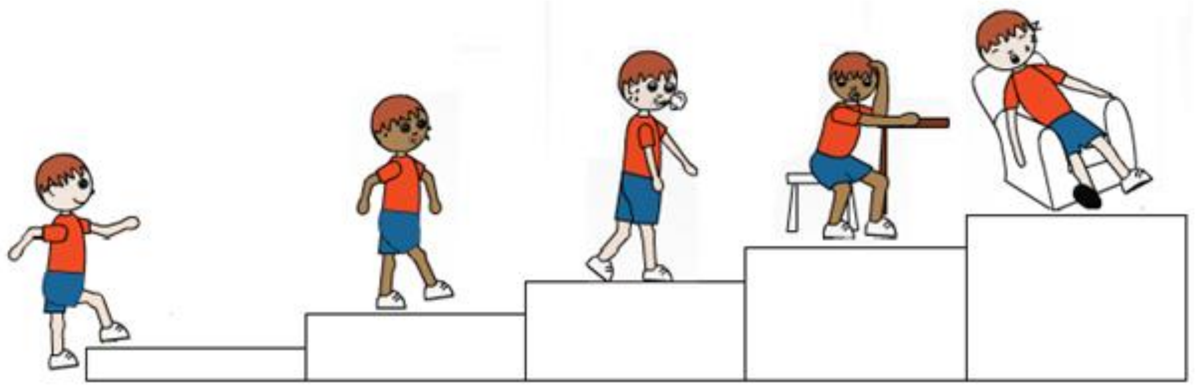

Kodi mu na mvelako ku chepekela mphamvu?

Ndi bvomekeza ☐ Sindi bvomekeza ☐

Kodi mu na onewako ndi a dotolo amene ayanganila pa matenda olinga ku za mphamvu ndi malungo?

Ndi bvomekeza ☐ sindi bvomekeza ☐

kodi ana kupezankoni ndi bvuto la kubvimba malungo {bvuto la mu malungo ndi mphamvu ?

Ndi bvomekeza ☐ Sindi bvomekeza ☐

Ngati nitelo, ndi kufufuzidwa ko tani kuna chitisidwa? Tikupemphani kuti muti masulile ngati za magazi ndi zo kopedwa mukati mwa thupi.

Nanga kuli wina mu banja lanu a mene ana pezedwa ndi bvuto la ku bvimba mu malungo?

Ndi bvomekeza ☐ Sindi bvomekeza ☐

Ngati nitelo, nidani, ndipo ndi bvuto lotani?
