# Supplementary material for: Impact of musculoskeletal symptoms on physical functioning and quality of life among treated people with HIV in high and low resource settings: A case study of the UK and Zambia
Source: PLoS One. 2019 May 13;14(5):e0216787. doi: 10.1371/journal.pone.0216787 (PMC6513081; doi:10.1371/journal.pone.0216787)
Supplement: S7 File — (PDF) [file pone.0216787.s007.pdf]

Questionnaire:

Functional Capacity using the Health Assessment Questionnaire

tiku pemphani kuti mu chonge ku langiza mwa mene mu na kwanisila pa kuchita zinthu zo siana pa sabatha la pita.

	kopanda bvuto iliyonse	Ndi bvuto la pa ng'ono	Kopanda bvuto la likulu	kulepela ku chita
1. Kubvala ndi ku konzekela				
Kodi mukwanisa ku:				
a. Zivalika ndi ku manga nthambo za nsapato ndi ku manga ma batani	☐	☐	☐	☐
b. ku sambika mu mutu	☐	☐	☐	☐
2. Kuima				
Kodi munga kwanise ku:				
a. ku nyamuka pa mu pando ulibe manja}	☐	☐	☐	☐
b. ku lowa ndi ku nyamuka po gona	☐	☐	☐	☐
3. Kudya				
Kodi munga kwanise ku:				
a. kujuwa nyama yanu	☐	☐	☐	☐
b. ku nyamula cup ya gilasi ndi kuika ku kamwa kanu	☐	☐	☐	☐
c. ku tsegula chi katundu cha mkaka kapena sopo lo chapila la unga	☐	☐	☐	☐

4. Kuyenda	kopanda bvuto iliyonse}	bvuto la pa ng'ono	bvuto la maningi}	kulepela ku chuita}
Kodi munga kwanise ku:				
a. Kuyenda pabwalo?	☐	☐	☐	☐
b. Kuyenda myenda zili asanu?	☐	☐	☐	☐
**5. Udongo**				
Kodi munga kwanise ku:				
a. Ku sambika ndi ku yumitsa thupi lako lonse?	☐	☐	☐	☐
b. Ku samba pa thupi?	☐	☐	☐	☐
c. Ku ngeni ndi ku choka mu chimbuzi?	☐	☐	☐	☐
**6. Kufika**				
Kodi munga kwanise ku:				
a. Ku tenga thumba la mbatatisi ili pa mutu panu ndi ku ika pansi	☐	☐	☐	☐
b. ku welama kuti ndi tenge nyula/nsalu ili pansi?	☐	☐	☐	☐
**7. Ku gwilisa**				
Kodi mu kwanitsa ku:				
a. Ku tsegula ziseko za gali moto?	☐	☐	☐	☐
b. Ku tsegula mbiya zimene zina tseguliwapo kale?	☐	☐	☐	☐
c. Ku vala ma pompi a madzi?	☐	☐	☐	☐

8. Zo chitika	kopanda bvuto iliyonse	Bvuto la pang'ono	Bvuto la li kulu	Kulepephela ku chita
Kodi unga kwanise ku;				
a. Za ma ulendo ndi malonda?				
b. Ku ngena ndi kuchoka mu gali moto?				
c. Ku gwila nchito za pa nyumba ngati; ku pyela mwa ku sewenzesa makina a malaiti, ndi ku samalila maluwa ndi mu munda?				

Tikupemphani kuti musankepo zili zonse zo thandizila zi mene mu ma sewenzesa pa zo chitika zo monga izi:

Ka mtengo

Ndodo

nsimbi zo thandizila kuti olemala kapena odwala kuti ayende bwino

ka mpando ka ma wilo awili ka mene odwala ama sewenzesa

Zinthu zo sewenzesa

Mpanda onyamuka mwamba

chi poto cho nkalapo pa ku chita chimbuzi

Mo sambila

Ka nsimbi ka mene ka ikidwapo zo sambila ngati nyula ndi zo pukutila thupi ndi zina zache

Zo sewenzesa zili ndi zogwilila zitali

Zo tsegulila mbiya

Zinthu zosewenzesa pa ku bvala

Zinthu zo sewenzesa pa ku bvala zobvala

Chulani zina:

Kulibe

Chongani pa malo aliyense amene mu ma funa thandizo ku chokela ku ena

ku zikonzekela mu mawa

ku uka

Udongo

kufika

Kulibe

kudya

kuyenda

kugwila zinthu ndi ku zi segula

Za ma ulendo ndi nchito za pa nyumba
