# Supplementary material for: Impact of musculoskeletal symptoms on physical functioning and quality of life among treated people with HIV in high and low resource settings: A case study of the UK and Zambia
Source: PLoS One. 2019 May 13;14(5):e0216787. doi: 10.1371/journal.pone.0216787 (PMC6513081; doi:10.1371/journal.pone.0216787)
Supplement: S8 File — (PDF) [file pone.0216787.s008.pdf]

Questionnaire:

SF- 36 Questionnaire – Quality of life

Please tick the one response which best describes your usual abilities and health

General Health

1. Pa zonse, kodi munga kambe kuti umoyo wanu uli otani?

Bwino maningi ☐ Bwino bwino ☐ bwino ☐ nkasako ☐ Sindili bwino ☐

2. kulinganidza ndi chaka chatha, kodi munga kambe kuti umoyo wanu unali otani?

Ndili bwino kupambana ndi chaka cha pita ichi ☐

Ndiliko nkasako kupambana chaka pita ichi ☐

Ndikali chi modzi modzi ☐

Ndili odwala ku pambana chaka cha chatha ☐

odwalilatu koposa chaka chatha ☐

Limitations of Activities

3. Zosatilapo ndi zo linga ku zimene munga chite mu kati mwa tsiku. Kodi umoyo wanu uma kulephelesani ku pezeka mu zinthu izi.

Ndi kwanisa movutikila Ndi kwanisa movutikilako Ndi kwanisa kopanda vuto

4. zi nchito za pakati, ngati ku sendeza tebulo, ku sewenzesa makina opyelela.

Ndi kwanisa movutikila Ndi kwanisa movutikilako Ndi kwanisa kopanda vuto

5. kunyamula zinthu zo sewenzesa mu nyumba ngati soap ndi zina zache zo gula ku ma stolo

Ndi kwanisa movutikila Ndi kwanisa movutikilako Ndi kwanisa kopanda vuto

6. Kuyenda kuwelenga myendo yangapo

Ndi kwanisa movutikila Ndi kwanisa movutikilako Ndi kwanisa kopanda vuto

7. Kuyenda kuwelenga myendo

Ndi kwanisa movutikila Ndi kwanisa movutikilako Ndi kwanisa kopanda vuto

8. ku welama, ku gwada kapena kuwelamila ku sogolo.

Ndi kwanisa movutikila Ndi kwanisa movutikilako Ndi kwanisa kopanda vuto

9. kuyenda mtunda utali.

Ndi kwanisa movutikila Ndi kwanisa movutikilako Ndi kwanisa kopanda vuto

10. kuyenda nchelwa zingono.

Ndi kwanisa movutikila Ndi kwanisa movutikilako Ndi kwanisa kopanda vuto

11. kuyenda nchelwa imodzi.

Ndi kwanisa movutikila Ndi kwanisa movutikilako Ndi kwanisa kopanda vuto

12. ku samba kapena ku dzivalika

Ndi kwanisa movutikila Ndi kwanisa movutikilako Ndi kwanisa kopanda vuto

Physical Health Problems

Pa ma sabatha anai apita, muna nkalako ndi mabvuto olinga ku ncthito yanu kapena ku zo chitika za tsiku ndi tsiku pa chifukwa cha umoyo wa ku thupi lanu?

13. ku chepetsako nthawi lo gwila ntchito ndi lo chita zinthu zina

    Ndibvomekeza Sindi bvomekeza

14. mu na kwanilisako chabe pa ng'ono ku lingana ndi muyeso mu nali kufuna

    Ndibvomekeza Sindi bvomekeza

15. Mu na pelewela pa nchito mu nali ku chita kapena pa zina zo chitika.

    Ndibvomekeza Sindi bvomekeza

16. Ndi nali ndi bvuto ku gwila ntchito ndi zina zo chitika.

    Ndibvomekeza Sindi bvomekeza

Emotional Health Problems

Pa ma sabatha anai apita, mwa nkalako ndi mabvuto aliyense olinga ku nchito lanu kapena ku zi mene zi ma chitika nthawi zonse chifukwa cha mabvuto a mu mtima kapena kuda nkawa

17. Ku chepetsako pa nthawi lo sewenza pa nchito kapena pa zina zo chitika

    Ndibvomekeza   Sindi bvomekeza

18. muna kwanisa pang'ono kulingana ndi muyeso , munali kufuna.

    Ndibvomekeza   Sindi bvomekeza

19. sindi na gwile nchito kapena zina zili zonse mo samalila nga ndi nali ku chitila nthawi zonse**.**

    Ndibvomekeza   Sindi bvomekeza

Social Activities

20. pa ma sabatha anai apita, nanga umoyo wanu wa sokonezedwa mo tani ku mbali la banja, anzanu, kapena ku ma gulu

Kunalibe	Pa'ngono	Pakati	kuwawitsa	Kuwawitsa maningi

21. Ndi ku wawa kwa bwanji kwa mene mwa nkala nako pa ma sabatha anai apita?

Kulibe	Bwino maningi	bwino	pakati	Ku wawitsa	Ku wawitsa maningi

22. pa ma sabatha anai apita, ndi ku wawa ko tani ku na sokoneza ntchito-ku onjezelapo nchito la mu nyumba ndi la panja pa nyumba?

kunalibe	Pa ng'ono	Pakati	Pa' ngo'ngo	Kwa maningi

Energy and Emotions

Ma funso aya a langana pa mwa mene munvelela ndi mwa mene zinthu zili pa ma sabatha anai apita. Pa funso iliyonse, pasani yankho imodzi imene ilangiza mwa mene munvelela.

23. kodi munvela kunkala ndi mphamvu maningi?

Nthawi zonse	Nthawi za mbili	Ka nthawi kataliko	Nthawi zina	Pa ka nthawi	Kulibe

24. kodi mwa nkalako wa kuda nkawa?

Nthawi zonse	Nthawi za mbili	Pa ka nthawi	Nthawi zina	Ka nthawi	Kulibe

25. Kodi mu na mvelapo osakondwela kufikila kuti kulibe chi na koza ku ku kondwelesani?

Nthawi zonse	Nthawi za mbili	Pa ka nthawi	Nthawi zina	Ka nthawi	Kulibe

26. kodi mu na mvelako ku deka mtima ndi mtendere?

Nthawi zonse	Nthawi za mbili	Pa ka nthawi	Nthawi zina	Ka nthawi	Kulibe

27. kodi muli ndi mphamvu za mbili?

Nthawi zonse	Nthawi za mbili	Pa ka nthawi	Nthawi zina	Ka nthawi	Kulibe

28. kodi mwanvelako ngati muli ndi chikonko/okumudisidwa kapena osakondwela?

Nthawi zonse	Nthawi za mbili	Pa ka nthawi	Nthawi zina	Ka nthawi	Kulibe

29. kodi mu na mvelako kulema ko sili lathu nchito?

Nthawi zonse	Nthawi za mbili	Pa ka nthawi	Nthawi zina	Ka nthawi	Ka nthawi

30. kodi mwa nkalapo okondwela

Nthawi zonse	Nthawi zonse	Pa ka nthawi	Nthawi zina	Ka nthawi	Ka nthawi

31. Nanga Mwa mvelapo ku lema}

Nthawi zonse	Nthawi zonse	Pa ka nthawi	Nthawi zina	Ka nthawi	Ka nthawi

Social Activities

32. pa ma sabatha anai apita, ndi nthawi la itali bwanji pa mene umoyo olinga ku thupi kapena pa mabvuto a mu maganizo ana sokoneza pa zolinga ku tandala ndi anzanu ndi alendo,

Nthawi zonse	Nthawi zonse	Pa ka nthawi	Nthawi zina	Ka nthawi	Ka nthawi

General Health

Nenani ngati ndi zoona kapena ndi boodza pa mau osatilapo aya?

33. Ndi ma dwala mwa pafupi pafupi kusiyana ndi anthu ena.

Ndi cha zoonadi	Ndi cha zoona	sindiziwa	Ndi boza kambili	Ndi bozadi

34. Ndili ndi umoyo wa thanzi monga wina aliyense amene ndi dziwa

Ndi cha zoonadi	Ndi cha zoona	sindiziwa	Ndi boza kambili	Ndi bozadi

35. Ndi yembekezela umoyo wanga kudwala kopambana apa.

Ndi cha zoonadi	Ndi cha zoona	sindiziwa	Ndi boza kambili	Ndi bozadi

36. umoyo wanga uli bwino

Ndi cha zoonadi	Ndi cha zoona	sindiziwa	Ndi boza kambili	Ndi bozadi
