# Supplementary material for: Impact of musculoskeletal symptoms on physical functioning and quality of life among treated people with HIV in high and low resource settings: A case study of the UK and Zambia
Source: PLoS One. 2019 May 13;14(5):e0216787. doi: 10.1371/journal.pone.0216787 (PMC6513081; doi:10.1371/journal.pone.0216787)
Supplement: S9 File — (PDF) [file pone.0216787.s009.pdf]

Questionnaire:

Rheumatological symptoms:

The following questions refer to things that may have happened since you found out that you had HIV infection. Please tick the appropriate boxes.

Have you had pains in your joints? Yes ☐ No ☐

If so can you rate the severity using the pain rating scale?

No pain	0	1	2	3	4	5	6	7	8	9	10	Worst pain

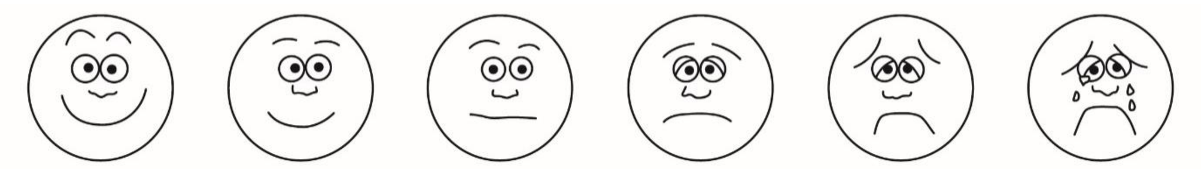

If so, can you please mark on the drawings below, the areas where you feel pain?

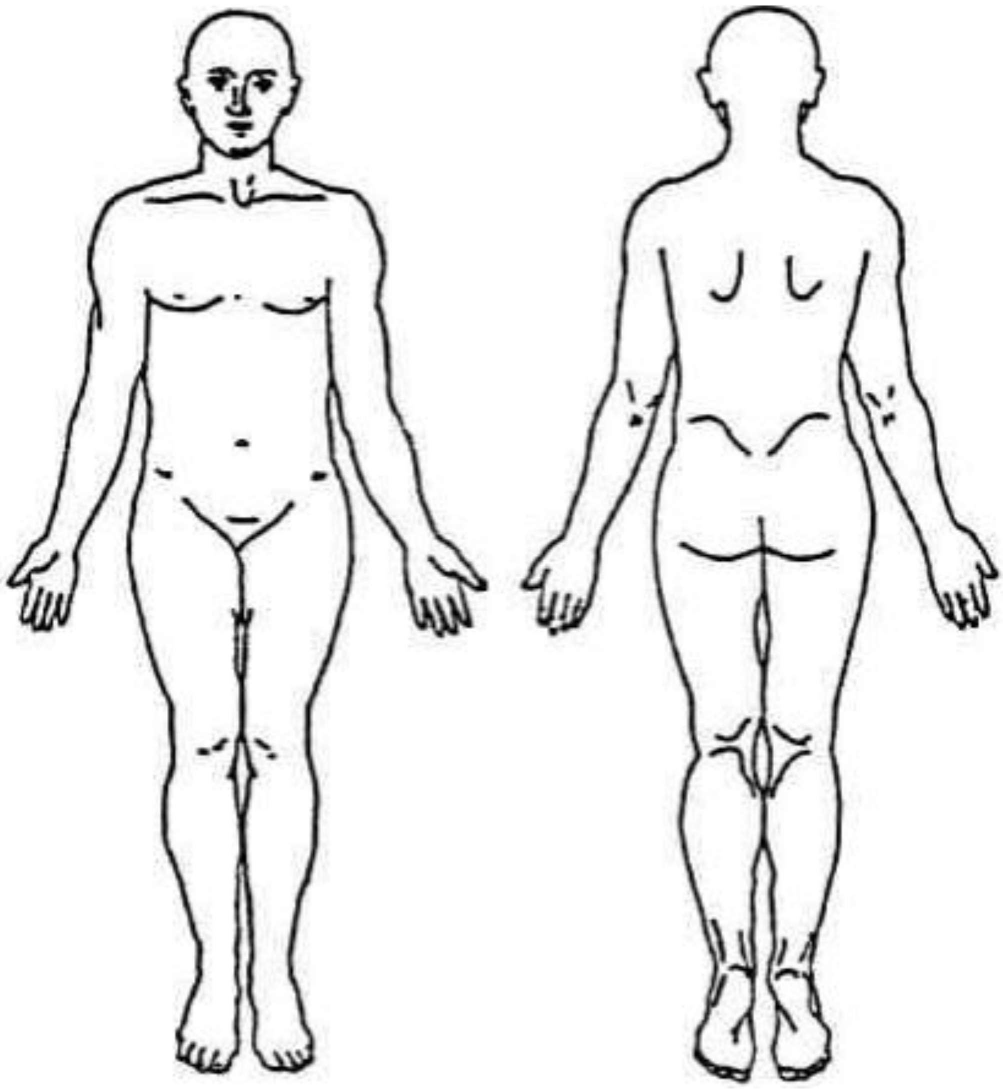

Have you had a swollen red joint? Yes ☐ No ☐

Have you noticed reduced joint movement? Yes ☐ No ☐

Do you have any joint or muscle stiffness in the morning? Yes ☐ No ☐

(By stiffness we mean when it is difficult to move your muscles and bend your joints; they are rigid or firm)

How long does your morning stiffness last from the time you wake up?

Less than 30 minutes ☐ More than 30 minutes ☐

Have you had pains in your muscles? Yes ☐ No ☐

If so can you rate the severity using the pain rating scale?

No pain	0	1	2	3	4	5	6	7	8	9	10	Worst pain

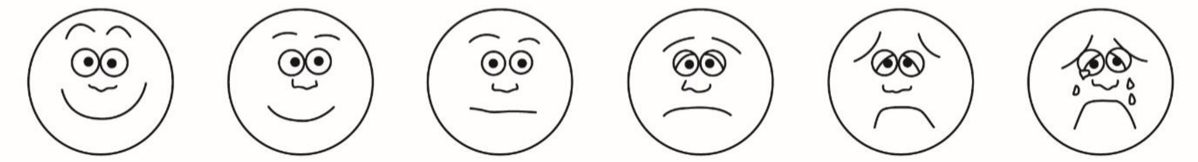

Have you had any neck pain or stiffness? Yes ☐ No ☐

Have you had any back pain or stiffness? Yes ☐ No ☐

Have you noticed any pain in your heels? Yes ☐ No ☐

Have you had difficulty in taking or gripping/squeezing something? Yes ☐ No ☐

Do your eyes persistently burn, feel dry or gritty? Yes ☐ No ☐

Have you had any sores in your mouth? Yes ☐ No ☐

Do your hands turn white or blue on exposure to cold? Yes ☐ No ☐

Have you had any skin rash? Yes ☐ No ☐

If so, can you describe the rash?

Have you noticed any changes in your nails? Yes No

Have you noticed any discomfort when passing water? Yes No

Have you had any difficulty sleeping? Yes No

Have you felt fatigued? Yes No

If so, can you rate the severity using the below rating scale?

Not fatigued	0	1	2	3	4	5	6	7	8	9	10	Extremely fatigued

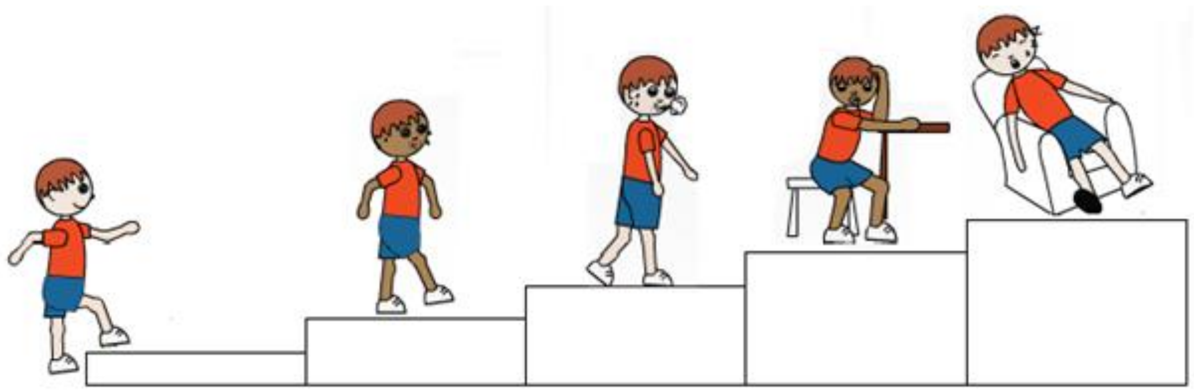

0 1 2 3 4 5 6 7 8 9 10

Have you felt weak? Yes No

Have you ever seen a Rheumatologist (A doctor specialised in diseases of the muscles and joints)?

Yes No

Have you ever been diagnosed with a rheumatological condition? Yes No

What investigations were carried out? Please give details e.g. blood tests, x-rays:

Has anyone else in the family ever been diagnosed with a rheumatological condition?

Yes ☐ No ☐

If so, who and with what condition?
