# Supplementary material for: Impact of musculoskeletal symptoms on physical functioning and quality of life among treated people with HIV in high and low resource settings: A case study of the UK and Zambia
Source: PLoS One. 2019 May 13;14(5):e0216787. doi: 10.1371/journal.pone.0216787 (PMC6513081; doi:10.1371/journal.pone.0216787)
Supplement: S10 File — (PDF) [file pone.0216787.s010.pdf]

Socio-demographic data:

Patient Number:

Demographics:

Male ☐ Female ☐

Age ☐ Age at diagnosis: ☐ Years until diagnosis: ☐

Education level: None ☐ Elementary ☐ College ☐ University ☐

Income level (per annum): ≤ £20 000 ☐ £20 000 – 30 000 ☐ £30 000 – 40 000 ☐

≥ £50 000 ☐

Marital status: Married ☐ Single ☐ Partnership ☐

Work status: Employed ☐ Unemployed ☐

Occupation:

Are you the earning member of the family: Yes ☐ No ☐

Religion:

Smoker: Yes ☐ No ☐ BMI (height & weight):

Route of HIV transmission: Heterosexual sex ☐ Homosexual sex ☐ Blood products ☐

Vertical transmission ☐

Co-morbidities (do you have any other medical, surgical or psychological problems)

Medical	
Surgical	
Psychological	

Do you have?

Hepatitis B: Yes ☐ No ☐

Hepatitis C: Yes ☐ No ☐

Tuberculosis: Yes ☐ No ☐

Other sexually transmitted infections: Yes ☐ No ☐

If so, which ones? Please provide details below e.g. diagnosis, management:

Number of hospital admissions since diagnosis: ☐

Please give details of each hospital admission e.g. dates, diagnoses, management

Investigations:

Most recent CD4 count: ☐

Most recent viral load: ☐

Management:

Are you taking anti-retroviral therapy? Yes ☐ No ☐

What regimen of anti-retroviral therapy are you taking?

Please give details e.g. dates, dose:
